# Measuring the Benefits of Healthcare: DALYs and QALYs – Does the Choice of Measure Matter? A Case Study of Two Preventive Interventions

**DOI:** 10.15171/ijhpm.2017.47

**Published:** 2017-05-06

**Authors:** Federico Augustovski, Lisandro D. Colantonio, Julieta Galante, Ariel Bardach, Joaquín E. Caporale, Víctor Zárate, Ling Hsiang Chuang, Andres Pichon-Riviere, Paul Kind

**Affiliations:** ^1^Institute for Clinical Effectiveness and Health Policy (IECS-CONICET), Buenos Aires, Argentina.; ^2^University of Alabama at Birmingham, Birmingham, AL, USA.; ^3^Cardiff University, Cardiff, UK.; ^4^Facultad de Medicina, Universidad San Sebastian, Santiago, Chile.; ^5^Pharmerit, Rotterdam, The Netherlands.; ^6^University of Leeds, Leeds, UK.

**Keywords:** Quality-Adjusted Life Year (QALY), Disability-Adjusted Life Year (DALY), Health Benefit Measure, Economic Evaluation

## Abstract

**Background:** The measurement of health benefits is a key issue in health economic evaluations. There is very scarce empirical literature exploring the differences of using quality-adjusted life years (QALYs) or disability-adjusted life years (DALYs) as benefit metrics and their potential impact in decision-making.

**Methods:** Two previously published models delivering outputs in QALYs, were adapted to estimate DALYs: a Markov model for human papilloma virus (HPV) vaccination, and a pneumococcal vaccination deterministic model (PNEUMO). Argentina, Chile, and the United Kingdom studies were used, where local EQ-5D social value weights were available to provide local QALY weights. A primary study with descriptive vignettes was done (n = 73) to obtain EQ-5D data for all health states included in both models. Several scenario analyses were carried-out to evaluate the relative importance of using different metrics (DALYS or QALYs) to estimate health benefits on these economic evaluations.

**Results:** QALY gains were larger than DALYs avoided in all countries for HPV, leading to more favorable decisions using the former. With discounting and age-weighting – scenario with greatest differences in all countries – incremental DALYs avoided represented the 75%, 68%, and 43% of the QALYs gained in Argentina, Chile, and United Kingdom respectively. Differences using QALYs or DALYs were less consistent and sometimes in the opposite direction for PNEUMO. These differences, similar to other widely used assumptions, could directly influence decision-making using usual gross domestic products (GDPs) per capita per DALY or QALY thresholds.

**Conclusion:** We did not find evidence that contradicts current practice of many researchers and decision-makers of using QALYs or DALYs interchangeably. Differences attributed to the choice of metric could influence final decisions, but similarly to other frequently used assumptions.

## Background

The measurement of health benefits is a key issue in health economic evaluations and health policy, however the choice of the metric of measurement is not uniformly acknowledged. Traditional cost-benefit measurement, for example, requires that both costs and consequences are measured in terms of monetary value. Whilst the measurement of costs is relatively straightforward, there are differing opinions as to the mechanism for assigning a monetary value to health benefits – and indeed whether such an assignment is acceptable. For the measurement of health benefit to have the greatest potential use in order to inform allocative efficiency decisions, it needs to be based on a generic system so that gains/losses can be compared across the widest possible range of therapeutic interventions. In this context, measures of health or health assessment, and specially combined metrics that incorporate life expectancy and quality of life or disability have been widely adopted. Two main different generic paradigms have been proposed for setting healthcare priorities: quality-adjusted life years (QALYs) based on the effect of interventions, and disability-adjusted life years (DALYs) based on the burden of disease in a population. These formal paradigms recall the assumptions implicitly made in the everyday delivering of, and hence rationing access to, healthcare. Nowadays, these two paradigms have emerged as the main contenders in informing national agencies and global decision-makers, although not without controversy and with the availability of other alternative potential metrics.^1^ Due to the scarcity of empirical comparisons of economic evaluations using QALYs or DALYs, we undertook a comparative exploratory exercise based on recent economic evaluations our group was involved in. In order to have a broad scope, we included economic evaluations based on two models evaluating two vaccines (human papilloma virus - HPV - and conjugated pneumococcal) with researchers from the collaborative team.

## Methods

Two preventive models were selected in order to empirically address the effect of using DALY and QALY-based methods to compute health benefits in economic evaluations: an HPV cohort based Markov vaccination model,^2^ and a pneumococcal compartmental vaccination model.^3^ Both models were developed in Microsoft Excel^®^, and originally used only QALYs as the primary health benefit measure, incorporating QALY weights from international sources.

These models have been applied in different countries around the world.^2,4,5^ For the present study, we analyzed and compared model adaptations from Argentina, Chile, and the United Kingdom because EQ-5D social value weights were available for these countries.^6-8^ This allowed to explore the impact of country-specific utility weights for QALYs on results across countries. In addition, models were reprogrammed in order to estimate both components required to estimate DALYs: years of life with disability (YLD) produced by the same conditions, as well as the years of life lost (YLLs) by premature death. See more details about model description in Appendix 1.

### Human Papilloma Virus Vaccination Model

The HPV vaccination model is a Markov model reflecting the natural history of oncogenic HPV infection, through 12 different health states. The model follows a cohort of 11-year old girls over lifetime under two different strategies: current Pap screening program or current Pap screening program plus bivalent HPV vaccination at 12 years old. General description of the model and results for Argentina, Chile, and United Kingdom has been published elsewhere.^2,5^ Briefly, the model considers the epidemiology of HPV, the characteristics of the screening program, the treatment guidelines and the specific costs for each country. In order to model the natural history of oncogenic HPV infection and cancer development, incidences of HPV infection for each country were modeled from local prevalence studies. For all other state-transition probabilities, as local studies were not available, data from the United Kingdom was used for all three countries. Finally, models were calibrated to country specific vital statistics in order to reproduce local epidemiology of HPV related diseases.

### Pneumococcal Vaccination Model

The pneumococcal vaccination model is static, deterministic and age-compartmental.^3^ It considers the occurrence of four pneumococcal related diseases (meningitis, bacteraemia, pneumonia and acute otitis media) in a calendar year, across all age cohorts. Additionally, present value of future costs and disutilities related to events’ sequelae that start in that year are incorporated. The model is able to address the cost-effectiveness of the introduction of different pneumococcal vaccines (Synflorix^®^ or Prevnar^®^ as compared to no vaccination) in local vaccination programs. In the present study we reprogrammed the model using Synflorix^®^ as an example.

### Estimating Quality-Adjusted Life Years

For each health state in both models, descriptive vignettes based on the EQ-5D-3L instrument were administered to a convenience sample of 73 healthy people in Argentina who had completed their high school studies, in order to gather descriptive health state data. More details of this sub-study were previously published.^9^ Finally, as local social values are available in the three countries analyzed,^6-8^ these country specific EQ-5D weights were used in order to map the same mix of descriptive health states for each health state into local QALY weights.

Pneumococcal vaccination model also contemplates including normative utilities by age, that is, the mean utility of spending a year alive for the general population. Normative utilities were obtained from local valuation studies and population surveys of Argentina,^6,10^ Chile (V. Zarate V, P. Kind, personal communication, 2009)^8,11^ and the United Kingdom.^11^ In a scenario analysis we explored the impact of using QALYs estimated without normative utilities.

### Calculating Disability-Adjusted Life Years

By default, both models estimate QALYs, so they were reprogrammed in order to include DALYs. To calculate DALYs we employed individual equations for YLL and YLD published elsewhere.^12-15^ A population figure was obtained by multiplying each individual result by the incident cases, deaths or disability cases that were estimated by the models. Additional details about the DALY calculations can be found in Appendix 2.

Standard expectation of life for YLL calculations were extracted from life-table West Level 26.^14^ In an alternative scenario we used local life tables for each country. Disability weights were obtained from the 2004 update of the Global Burden of Disease study^12,16^ for all the pneumococcal vaccination model states. Disability weights unavailable for the HPV vaccination model (ie, all states except cancer related states), were obtained following the methodology described in a Dutch study.^15^ This study transformed EQ-5D descriptive data to a disability weight using a multiple regression equation. There was no need to estimate independently disease duration for estimating DALYs (ie, with DISMOD model) as they were obtained by the Markov models.

### Models Analysis

For the analysis, epidemiological parameters (such as incidence, acute mortality, or transition between different states) were kept as in the original published studies.^2,3^ The main difference in the base-case analysis from the original studies were: (1) that specific EQ-5D values using country specific value sets for health state were included; and (2) models have been reprogrammed to estimate DALYs alongside QALYs based on GBD weights as described. For both models we selected to report the analysis of the QALY vs. DALY using a denominator of 100 000 subjects, though this was not always the denominator in the published studies.

First, we estimated the incremental benefit associated to each of the interventions, using both QALYs gained and DALYs avoided. Four scenarios to compare QALY with DALY results were estimated: with and without a 3.5% annual discount rate for both QALYs and DALYs, and with and without age weighting for DALYs. Second, in order to compare the relative difference between QALYs and DALYs in each scenario, a DALYs avoided/QALYs gained percentage ratio was estimated. So, a percentage ratio of 100% means that benefits were exactly the same with both metrics, a percentage ratio greater than 100% means that DALYs avoided were greater than QALYs gained, and a percentage ratio lower than 100% means that QALYs gained were greater. Third, we estimated the potential impact on decision-making of using these different metrics. In order to achieve this, for each model and country, we estimated the maximum incremental costs per person receiving the intervention that would make the strategy be deemed very cost-effective or cost-effective. Though now controversial, for illustration on how a specific decision rule, using either QALYs or DALYs, could impact the study conclusions, we used the willingness to pay thresholds according to gross domestic product (GDP) per capita proposed by the World Health Organization (WHO).^17^ According to this guidance, an intervention is very cost-effective if its incremental cost-effectiveness ratio (ICER) is less or equal 1 GDP per capita per DALY; cost-effective if it ranges from >1 and < equal 3; and not cost-effective if it is greater than three GDPs per capita per DALY. Though this rule was originally designed for DALYs, more commonly used in lower and middle income country settings, it is often used interchangeably for QALYs, used both in developed countries as well as in less developed ones. GDPs for each country were obtained from the International Monetary Fund and expressed in International Dollars 2013,^18^ which represent a Purchasing Power adjusted value.

Finally, in order to explore some potential factors that may influence these differences, we explored the impact of some model assumptions on several additional scenario analyses (sensitivity analysis). We explored replacing each countries EQ-5D based QALY weights by “1-DALY’s disability weight”; we varied influential parameters in the parent economic evaluation in the case of pneumococcal vaccine – those related to acute otitis media; used of local life expectancies to estimate DALYs; and incorporated normative (general population) utilities to estimate QALYs in the HPV vaccination model which were not incorporated in the base-case analyses; and also tested a scenario where the demographic characteristics of the three countries population was standardized in order to explore whether demographic differences had an impact in the QALY vs. DALY differences. The alternative scenario results are not the focus of the main paper; interested readers can see Appendix 3.

## Results

### Human Papilloma Virus Vaccination Model

Table 1 shows QALYs gained and DALYs avoided comparing HPV vaccination against no intervention in the four base-case scenarios (with and without discounting of both QALYs and DALYs; and with and without DALYs age-weighting). In most of the analyses, QALYs gains were larger than DALYs avoided, which would lead to more favorable decisions regarding cost-effectiveness if QALYs were used as the benefit metric.

Differences were larger in the United Kingdom and smaller in Argentina. The incorporation of discounting and DALYs age weighting affected most markedly the estimation of DALYs, increasing the difference with QALYs in all countries, where incremental DALYs avoided represented 75%, 68%, and 43% of the QALYs gained in Argentina, Chile, and the United Kingdom respectively. On the other hand, differences were smaller, and within +/- 20% range when no discounting nor age weighting were used.

**Figure 1:**
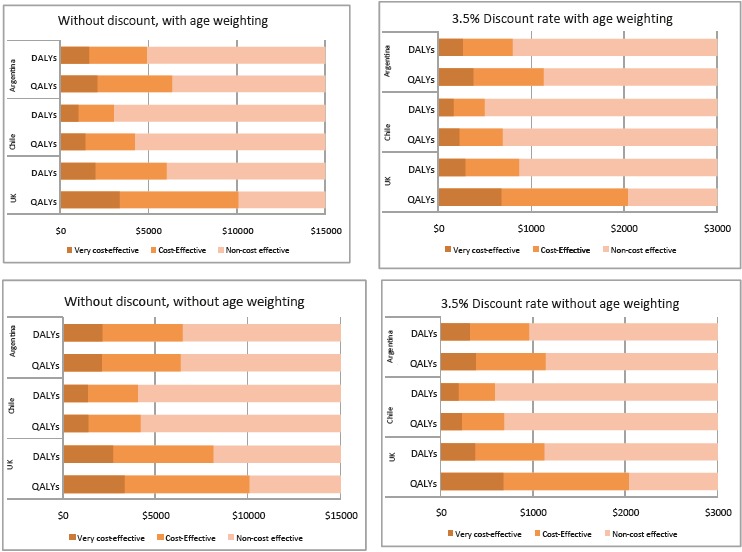
HPV Vaccination Strategy: Mean Incremental Costs Where Decisions Using 1 or 3 GDP Thresholds Change Using QALYs or DALYs for Argentina, Chile and the United Kingdom. Main scenarios for basal analysis (with and without discounting; with or without DALY age-weighting).
Abbreviations: QALYs, quality-adjusted life years; DALYs, disability-adjusted life years; GDP, gross domestic product; HPV, human papilloma virus.

In order to depict how these differences in the magnitude of benefit estimation if using QALYs or DALYs could have influenced decision-making, we show in Figure 1 how a new intervention with a particular incremental cost would change the decision based on QALYs or DALYs, and using the 1 and 3 GDP thresholds for QALYs or DALYs in the four scenarios and the three countries.

For instance, in the United Kingdom (with discounting and no age weights) as QALY estimation results in more benefits, an incremental cost per vaccinated person up to $2036 would be considered cost-effective using a 3 GDP threshold; while the same intervention would be considered cost-effective using DALYs only if the incremental cost per person is $1124 or less. This is another way of reflecting the 55% percentage ratio shown in Table 1 for this scenario. In this case example, decisions were more likely to agree if discounting and age weighting are used, but this can depend on the value of the final incremental cost of the study, and thus the incremental cost-effectiveness ratio for each country in relation to the decision threshold.

**Table 1:** Incremental Healthy Years (QALYs Gained or DALYs Avoided) Comparing HPV Vaccination Against no Vaccination (per 100 000 Women)^a^

**Country**	**QALYs Gained**	**DALYs Avoided**	**DALY/QALY Percentage Ratio**
**Without Discount, With Age Weighting on DALYs**			
Argentina	13 750.93	11 377.14	82.74%
Chile	10 034.62	6969.72	69.46%
UK	9189.99	5484.66	59.68%
**3.5% Discount Rate With Age Weighting on DALYs**			
Argentina	2458.03	1843.39	74.99%
Chile	1669.11	1142.28	68.44%
UK	1858.57	796.37	42.85%
**Without Discount, Without Age Weighting on DALYs**			
Argentina	13 750.93	14 965.23	108.83%
Chile	10 034.62	9321.46	92.89%
UK	9189.99	7431.34	80.86%
**3.5% Discount Rate Without Age Weighting on DALYs**			
Argentina	2 458.03	2 211.29	89.96%
Chile	1 669.11	1 357.41	81.33%
UK	1 858.57	1 025.83	55.19%

Abbreviations: QALY, quality-adjusted life year; DALY, disability-adjusted life year. ^a^ Main scenarios for basal analysis (with and without discounting; with or without DALY age-weighting).

### Pneumococcal Vaccination Model

Differences in incremental benefits using QALYs gained or DALYs saved show a different picture than in the HPV case study. In this case QALYs and DALYs gains were similar, and sometimes greater using DALYs, which would either not affect the decision or lead to more favourable decisions if DALYs were used as the benefit metric.

Table 2 shows incremental benefits in each country and scenario. Differences usually decreased when using discounting and no age weighting. Unlike the HPV case, in which differences were consistently larger in the United Kingdom and smaller in Argentina, there was no uniform pattern for all scenarios. Differences were larger in Argentina in those scenarios with age weighting, and larger in the United Kingdom in those scenarios without age weighting.

**Table 2:** Incremental Healthy Years (QALYs Gained or DALYs Avoided) Comparing Conjugated Pneumococcal Vaccination Against no Vaccination (Per 100 000 Subjects)^a^

**Country**	**QALYs Gained**	**DALYs Avoided**	**DALY/QALY Percentage Ratio**
**Conjugated Pneumococcal Vaccine vs. no Vaccination**			
**Without Discount, With Age Weighting on DALYs**			
Argentina	16.50	20.27	122.85%
Chile	35.84	40.10	111.88%
UK	21.47	22.29	103.83%
**3.5% Discount Rate With Age Weighting on DALYs**			
Argentina	6.95	7.25	104.38%
Chile	15.75	15.72	99.79%
UK	13.20	13.14	99.55%
**Without Discount, Without Age Weighting on DALYs**			
Argentina	16.50	19.22	116.50%
Chile	35.84	40.00	111.61%
UK	21.47	28.95	134.85%
**3.5% Discount Rate Without Age Weighting on DALYs**			
Argentina	6.95	6.87	98.94%
Chile	15.75	16.39	104.02%
UK	13.20	17.36	131.58%

Abbreviations: QALY, quality-adjusted life year; DALY, disability-adjusted life year. ^a^ Main scenarios for basal analysis (with and without discounting; with or without DALY age-weighting).

As an example, in the United Kingdom, QALY-DALY differences showed by the DALY/QALY percentage ratio were lower when age weighting was used in order to estimate DALYs avoided (difference ranged from -0.45% -percentage ratio 99.55%- [with discount and age weighting] to +34.85% [with age weighting and no discount]).

**Figure 2:**
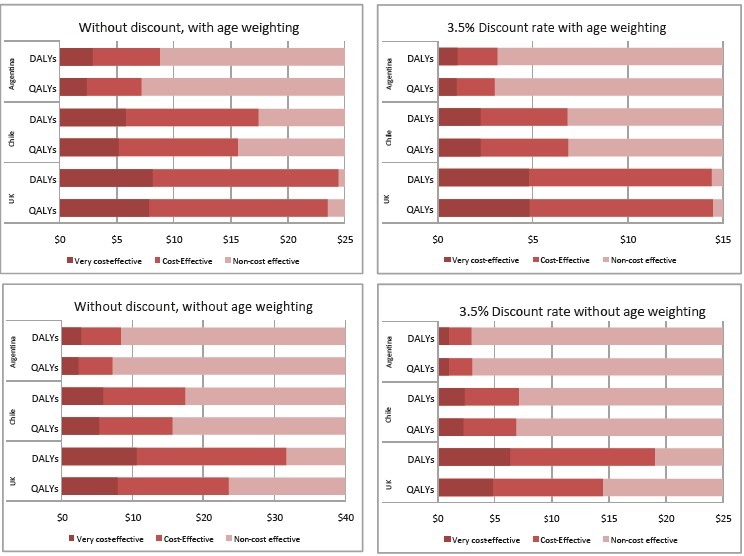
Pneumococcal Vaccination Strategy: Mean Incremental Costs Where Decisions Using 1 Or 3 GDP Thresholds Change Using QALYs or DALYs for Argentina, Chile and United Kingdom. Main scenarios for basal analysis (with and without discounting; with or without DALY age-weighting).
Abbreviations: QALYs, quality-adjusted life years; DALYs, disability-adjusted life years; GDP, gross domestic product; HPV, human papilloma virus.

The influence of these differences between QALYs and DALYs in decision-making was smaller than in the HPV case and is shown in Figure 2.

Similarly to Figure 1, Figure 2 depicts the mean incremental cost of the strategy at which the decision would change. Differences were rather small (within 5%) in the scenario with discounting and age weighting in all countries. In other scenarios, they were within +/-20%, except in the United Kingdom, where in the scenarios without age weighting DALYs were about 30% higher than QALYs. As an example, comparing conjugated pneumococcal vaccine strategy to no vaccination in Argentina (scenario without discounting and with age weighting) an incremental cost of the intervention of $8.5 would make the intervention cost-effective if DALYs are used, but cost-ineffective if QALYs are used instead.

In order to contextualize the findings and gauge the relevance of the benefit metric used versus other related choices researchers have when performing economic evaluations, we evaluated how, with each methodological choice, always compared to a common strategy – a scenario with discounted QALYs and normative utilities- the benefits can change. A summary of the influence of the different choices for both disease cases is shown in Table 3, where we show, for each of the researchers potential structural/methodological choices (columns) how much the baseline benefit differences between QALYs and DALYs change.

**Table 3:** Impact of Scenario Choice on the Estimation of the Magnitude of Benefit Difference, Expressed as the Proportion of Increase or Decrease From a Comparator Scenario of Using QALYs With Normative Utilities and Discounting of Both QALYs and DALYs

	**DALYs** **(No Age Weighting)**	**DALYs (With Age Weighting)**	**QALYs (No Discount)**	**QALYs (Non-normative Utilities)**	**1-DALY´s Disability Weight**
**HPV**					
Argentina	20%	3%	341%	31%	7%
Chile	15%	-1%	394%	38%	-5%
UK	-25%	-48%	439%	56%	0%
**Pneumococcal**					
Argentina	-1.1%	4.4%	139.0%	6.0%	-8.1%
Chile	3.9%	-0.2%	122.6%	13.4%	-13.2%
UK	24.0%	-0.3%	47.6%	17.9%	-5.0%

Abbreviations: QALYs, quality-adjusted life years; DALY, disability-adjusted life year; GDP, gross domestic product; HPV, human papilloma virus.

As we can see, some of the assumptions can influence the results similarly or more than the choice of QALYs or DALYs as the benefit metric. The largest differences were seen comparing to undiscounted results, which is not a practice currently recommended. In the case of HPV, the inclusion or exclusion of normative utilities was one of the most influential parameters, even more influential than the choice of the benefit metric (DALY or QALY). In the case of Pneumococcal disease, methodological choices showed the impact of discounting, and a lower impact of not using normative utilities. The other choices (DALYs with or without age weight, or using 1-DALY’s disability weight) had an inconsistent and heterogeneous influence in the differences.

More details about the results for the alternative analyses can be seen in Appendix 3.

## Discussion

There is no widespread agreement about the choice of the measure of health assessment in health economic evaluations, and there is also no consensus on using or not combined metrics -such as QALYs or DALYs. Germany, for example, favors natural units (ie, mm Hg for blood pressure reduction) instead of using a combined metric that tries to incorporate healthy life and thus morbidity and mortality.^19^ At the global level, key players such as the WHO promotes the use of the DALYs, mainly intended for evaluating and comparing disease burden across countries.^16^ In most jurisdictions where guidelines for economic evaluations are in place, QALY are usually preferred, though there is no consensus or empirical recommendations about whether one metric should be chosen over another.^20,21^ We can conceptualize this problem as one of structural uncertainty; ie, the assumptions that we incorporate into the decision problem, and whether these assumptions influence results and decision-making. This is only one component of global uncertainty, which also includes parameter uncertainty and heterogeneity.^22^

In the context of almost no literature empirically addressing this issue, we performed an exploratory case study analysis using two different models, that had been used in real studies, in order to address whether using QALY gained or DALY avoided to estimate incremental health benefits could affect the final estimation of benefit impact, and consequently the cost-effectiveness estimation and the coverage decision when adopting widely used decision rules related to the per capita GDP per unit of benefit.^17^ In these two case studies, we did not find a systematic pattern regarding the magnitude and direction of the differences between DALYs and QALYs. These differences were greater in the HPV case, and usually significantly smaller in the case of pneumococcal vaccine.

We found that the decisional impact was higher in the HPV than in the pneumococcal case; and results were sensitive to discounting as well as age weighting of DALYs. Though the latter is no longer recommended for DALYs estimation for disease burden, discounting is a standard practice in economic evaluation.^23-27^

Nevertheless, the magnitude of the structural uncertainty due to the metric choice was not necessarily greater than that due to other assumptions tested, usually based on explicit value choices and defended on normative or technical grounds. This was especially true in the case of incorporating normative utilities, which significantly influence the amount of QALYs gained, and thus the differences between QALYs and DALYs. The results obtained using normative utilities to calculate QALY gains were significantly lower than using a utility of 1 for the general population.

One possible explanation for the differences observed between the case studies analyzed (HPV model vs. pneumococcal model) is that the pneumococcal model incorporates normative utilities in order to estimate QALY gains in people without pneumococcal disease (therefore QALY gains are lower than if a utility = 1 is assumed for this healthy people). Since normative utilities are not considered in the HPV model in order to estimate QALYs gained, differences using both metrics could be higher than those observed in the main analysis.

Many studies evaluate and criticize the guiding principles of DALYs or QALYs, the influence of value choices, methodology, usefulness for developing countries, or arguments for their standardization. Nevertheless, we found no empirical studies that posed our study question. One study in the tuberculosis field discusses the pros and cons of using DALYs or QALYs but makes no empirical comparison between the methods.^28^ Airoldi and Morton^29^ published a conceptual paper comparing the potential benefit of a healthcare intervention measured in terms of QALYs vs. DALYs. There are many differences with our work, and is thus difficult to extrapolate findings between the two. They isolate the effect of framing the problem from a health or a disability perspective; did not use age-weighting function in calculating DALYs, employed a common discounting methodology and the same set of quality of life and disability weights. They found that the main difference between these measures is the use of life expectancy tables to determine the years of life lost component of DALYs. Particularly, the use of a death-dependent reference age is problematic when the intention is to evaluate the impact of life saving interventions. In these cases, some authors recommended the use of local period life expectancy tables for single-year interventions ‘as long as the changes caused by the intervention do not change age-specific and overall life expectancies substantially.’^30^

Using our results for the HPV vaccination model showed that DALYs avoided were generally less than QALYs gained; just as Airoldi and Morton would predict for a life extending intervention. On the other hand, with the model that evaluated the introduction of conjugated Pneumococcal vaccination, another life extending intervention, we found the opposite; that is, DALYs avoided were generally more than QALYs gained.

Some limitations of our study should be noted. Results obtained from this exploratory study are difficult to extrapolate to other studies. We evaluated only two preventive interventions. As a case study, it was built on adapting previously used models, and not intended to generalize the findings to wider contexts. Also, there has been a recent update of disability weights for DALYs.^16^ Though the agreement between the newer and older disability weights set was high (r = 0.70) and would not probably alter significantly our results, this is an issue worth exploring in future studies.

## Conclusion

Our study, which adapted two previously published models to report health benefits both in QALYs and DALYs, shows that the magnitude of benefit could be significantly different when using one or the other, and these differences did not systematically favoured one metric vs. the other. The magnitude of this source of uncertainty was similar to that of other sources of uncertainty that could lead to different decisions about the cost-effectiveness of a health technology. As an empirical methodological case study, we consider it more as hypothesis generating that an explanatory study. We did not find evidence that contradicts current practice of many researchers and decision-makers of using and interpreting results of QALY or DALY based studies interchangeably and thus with the same decision-making threshold. A more generalizable analysis using specifically designed models could be necessary in order to be able to better understand the factors that explain the differences of using each metric.

## Ethical issues

This study was based on secondary models analyses and considered exempt for an Internal Review Board submission.

## Competing interests

The authors declare that they have no competing interests. This research and its manuscript were financed by an unrestricted and independent grant from GlaxoSmithKline Biologicals. The funders had no role in study design, data collection and analysis, or preparation of the manuscript.

## Authors’ contributions

FA, LDC, APR, and PK participated in the conception and design of the study; FA, LDC, and APR in the data analysis plan; LDC, JG, FA, and APR performed the analysis and interpretation of results; LDC, JG, AB, and JEC participated in the management and input collection for models. All authors participated in writing the article, in his critical review and final approval.

## Authors’ affiliations

^1^Institute for Clinical Effectiveness and Health Policy (IECS-CONICET), Buenos Aires, Argentina. ^2^University of Alabama at Birmingham, Birmingham, AL, USA. ^3^Cardiff University, Cardiff, UK. ^4^Facultad de Medicina, Universidad San Sebastian, Santiago, Chile. ^5^Pharmerit, Rotterdam, The Netherlands. ^6^University of Leeds, Leeds, UK.

## Key messages

**Implications for policy makers**

- Quality-adjusted life years (QALYs) and disability-adjusted life years (DALYs) are usually used interchangeably in economic evaluations of health technologies.
- This paper shows that the magnitude of health benefit estimated through QALYs or DALYs could be significantly different and potentially alter the study conclusions.
- The magnitude of the uncertainty associated to the choice of metric was not greater to other common sources of uncertainty in economic evaluations (ie, discounting, parameter or structural uncertainty).

**Implications for public**

In this paper we show that the choice of the measure of health benefit (Quality-adjusted life years [QALYs] or disability-adjusted life years [DALYs]) can potentially alter the results and conclusions of an economic evaluation to determine the cost-effectiveness of a health technology. This finding, together with other sources of uncertainty should be taken into account when designing, performing or reporting this type of studies.

## Appendix 1

### Appendix 1. Model Descriptions: Parameters and Assumptions

**Table:** Table A1. List of States Considered in the HPV Vaccination Model and Utilities Assumptions

**State**	**Description and Utilities**
No oncogenic HPV infection	Healthy woman. No disutility was assumed. A QALY weight of 1 was used in order to estimate total QALYs. No additional YLDs by this condition were assumed.
Oncogenic HPV infection	Asymptomatic condition. A same utility/disutility that a healthy woman was assumed.
CIN 1 undetected	Asymptomatic condition. A same utility/disutility that a healthy woman was assumed.
CIN 1 detected	Country specific QALY weights were estimated. Models were reprogrammed in order to measure YLDs by this condition.
CIN 2 & 3 undetected	Asymptomatic condition. A same utility/disutility that a healthy woman was assumed.
CIN 2 & 3 detected	Country specific QALY weights were estimated. Models were reprogrammed in order to measure YLDs by this condition.
Persistent CIN 2 & 3 undetected	Asymptomatic condition. A same utility/disutility that a healthy woman was assumed.
Persistent CIN 2 & 3 detected	A same utility/disutility that the CIN 2 & 3 detected state was assumed.
Cancer	Country specific QALY weights were estimated. Models were reprogrammed in order to measure YLDs by this condition.
Cancer cured	Country specific QALY weights were estimated. Models were reprogrammed in order to measure YLDs by this condition.
Overall death	A QALY weight of zero was use for this state. Models were reprogrammed in order to measure YLLs by premature death.
Death by cervical cancer	A QALY weight of zero was use for this state. Models were reprogrammed in order to measure YLLs by premature death.

Abbreviations: QALY, quality-adjusted life year; HPV, human papilloma virus; YLD, years of life with disability; YLL, years of life lost; CIN, cervical intraepithelial neoplasia.

**Table A2:** Health Conditions Considered in the Pneumococcal Vaccination Model^a^

**Disease Outcome**	**Duration (wk)**
Short-term disability associated with acute disease (applied to current year, not discounted)	
Meningitis (inpatient)	2.3143
Bacteraemia (inpatient)	1.7714
Pneumonia (inpatient)	1.5806
Pneumonia (outpatient)	0.7903
AOM (outpatient)	0.1429
AOM hospitalized myringotomy	0.1429
Long-term disability associated with sequelae (applied to lifetime, discounted)	
Neurological sequelae from meningitis	More than 1 year
Hearing Loss from meningitis	More than 1 year
Hearing Loss from AOM	More than 1 year

Abbreviation: AOM‏, acute otitis media. ^a^ Values correspond to the mean duration of disability for mentioned conditions according to the original health economic evaluation of pneumococcal vaccination for the United Kingdom.

## Appendix 2

### Appendix 2. DALYs Estimation Methods

Both models were reprogrammed in order to estimate DALYs. For calculating DALYs we followed a standard methodology described to estimate both YLDs and YLLs.^14^ For YLDs, we used the following equation:

$$\begin{array}{l} YLD=D*((\frac{K*C*e^{r*a}}{{\left(r+\beta \right)}^{2}})\\ {}*(e^{-(r+\beta )*(L+a)}*(-(r+\beta )*(L+a)-1)-e^{\begin{array}{l} (r+\beta )*a \end{array}}\\ {}*((-(r+\beta )*a-1))+\left(\frac{1-K}{r}\right)*(1-e^{\begin{array}{l} -r*L \end{array}})) \end{array}$$

where K = age weighting modulation factor (between 0 and 1); C = constant (0.1658); r = discount rate; a = age of onset of disability; β = parameter of the age weighting function (0.04); L = duration of disability; D = disability weight. A correction of this equation has to be made in order to prevent error when r = 0:

$$\begin{array}{l} YLD=D*((\frac{K*C*e^{\beta *a}}{\beta^{2}})*(e^{-\beta *L}*(-\beta )*(L+a)-1)-(-\beta *a-1))\\ +(1-K)*L) \end{array}$$

For YLLs, we used the following equation:

$$\begin{array}{l} \text{YLL = }\left(\frac{K*C*e^{r*a}}{{\left(r+\beta \right)}^{2}}\right)\\ {}*(e^{-(r+\beta )*(LE+a)}*(-(r+\beta )*(LE+a)-1)-e^{-(r+\beta )*a}\\ {}*(-(r+\beta )*a-1))+\left(\frac{1-K}{r}\right)*(1-e^{-r*LE})\\ \; \end{array}$$

where K = age weighting modulation factor (between 0 and 1); C = constant (0.1658); r = discount rate; a = age of death; β = parameter of the age weighting function (0.04); LE = standard expectation of life at age a. A correction of this equation has to be made in order to prevent error when r = 0:

$$\begin{array}{l} \text{YLL = }\left(\frac{K*C*e^{\beta *a}}{\beta^{2}}\right)*(e^{-\beta *LE}*(-\beta *(LE+a)-1)\\ -(-\beta *a-1))+(1-K)*LE\\ \; \end{array}$$

In all cases, a β value of 0.04 was used, according to Fox-Rushby.^14^ K and r values can be set as independent parameters in models.

Two parameters are especially important in order to estimate YLDs: duration of the condition, and disability weight associated. For the HPV vaccination model, duration of each health state by age of onset for women with different participation on screening program (regular, irregular or no participation) was measured from the same in order to not include systematic differences with QALYs estimation. For DALYs estimations, mean duration data was linked with the estimation cells, so this information is automatically taken from the performance of the model and was not considered as an input.

For the pneumococcal vaccination model duration of conditions that last less of 1 year was considered the same that the duration in the model, and were obtained from the health economic evaluation for the United Kingdom. In the other hand, conditions that last more than 1 year (auditive and neurologic sequelae) are assumed not to increase the probability of death and not to be reversible. So duration is equal to life expectancy (in each country) at age of onset (same assumption that in the health economic evaluation for the United Kingdom). The same assumption was used on QALY estimation. Standard methodology for DALYs estimation requires using the same disability weight for each country. The GBoD project covers a wider range of conditions, and disability weights (2004) provided has been widely used.^13^ As some of the weights are not included in the GBoD project, disability weights for stages of the HPV vaccination model were obtained using the same methodology described in the Dutch study, involving the estimation of weights for 175 disease stages, sequelae and severity levels. This method allows to estimate disability weights from EQ-5D descriptive states by mean of a regression model.^16^ EQ5D descriptive states were obtained from the survey mentioned previously and already published.^9^

## Appendix 3

### Appendix 3. Analyses of Alternative Scenarios

In order to address the potential influence of local EQ-5D social values/tariffs in the base case differences we replaced the values for each country and using 1-disability weight from GBD project as the quality of life weight. In that way we used a same coefficient in all countries in order to estimate QALYs gained, which is the complementary value of disability weights used for DALYs estimation. This change has a different effect on incremental utility differences using QALYs gained and DALYs avoided: an increase on DALY/QALY percentage ratio of about 6% in Argentina, a decrease between 3% and 5% (without and with discount respectively) in Chile, and a negligible change in the United Kingdom. This makes more similar the differences observed in Argentina and Chile, meaning that differences between these countries could be explained at least in part by using different EQ-5D tariffs. Table A4 shows the new QALYs gained and DALYs avoided estimations and Figure A4 shows the effect on threshold analysis which was of the same magnitude as mentioned previously. Differences between QALYs gained and DALYs avoided were again lower when no discount (in both QALYs and DALYs estimations) and no age weighting (in DALYs estimation) were applied.

**Table A4:** Incremental Utility Comparing HPV Vaccination Against no Vaccination (Per 100 000 Women)^a^

**Country**	**QALYs Gained**	**DALYs Avoided**	**DALY/QALY Percentage Ratio**
**Without Discount, With Age Weighting**			
Argentina	14 693.51	11 377.14	77.43%
Chile	9702.73	6969.72	71.83%
UK	9195.49	5484.66	59.65%
**3.5% Discount Rate With Age Weighting**			
Argentina	2626.00	1843.39	70.20%
Chile	1586.17	1142.28	72.01%
UK	1860.70	796.37	42.80%
**Without Discount, Without Age Weighting**			
Argentina	14 693.51	14 965.23	101.85%
Chile	9702.73	9321.46	96.07%
UK	9195.49	7431.34	80.82%
**3.5% Discount Rate Without Age Weighting**			
Argentina	2626.00	2211.29	84.21%
Chile	1586.17	1357.41	85.58%
UK	1860.70	1025.83	55.13%

Abbreviations: QALY, quality-adjusted life year; DALY, disability-adjusted life year; HPV, human papilloma virus. ^a^ Alternative analysis QALY coefficients = 1-disability weight.

**Table A3:** Normative Utilities for Each Country

**Age Group**	**Argentina**	**Chile**	**UK**
< 16 years	0.95	0.88	0.91
16-24 years	0.95	0.88	0.91
25-34 years	0.94	0.87	0.91
35-44 years	0.92	0.84	0.88
45-54 years	0.90	0.79	0.85
55-64 years	0.87	0.70	0.79
65-74 years	0.83	0.66	0.78
≥ 75 years	0.76	0.59	0.73

Finally, we incorporated normative utilities into the HPV vaccination models. This change decreased the expected QALYs gained, increasing DALY/QALY percentage ratio in all countries (see Table A5). This was translated in change in most of the cases, with thresholds analysis estimation for DALYs higher than threshold analysis estimations for QALYs (see Figure A5).

**Table A5:** Incremental Utility Comparing HPV Vaccination Against no Vaccination (Per 100 000 Women)^a^

**Country**	**QALYs Gained**	**DALYs Avoided**	**DALY/QALY Percentage Ratio**
**Without Discount, With Age Weighting**			
Argentina	9312.50	11 377.14	122.17%
Chile	6500.03	6969.72	107.23%
UK	5788.85	5484.66	94.75%
**3.5% Discount Rate With Age Weighting**			
Argentina	1778.19	1843.39	103.67%
Chile	1149.87	1142.28	99.34%
UK	1286.60	796.37	61.90%
**Without Discount, Without Age Weighting**			
Argentina	9312.50	14 965.23	160.70%
Chile	6500.03	9321.46	143.41%
UK	5788.85	7431.34	128.37%
**3.5% Discount Rate Without Age Weighting**			
Argentina	1778.19	2211.29	124.36%
Chile	1149.87	1357.41	118.05%
UK	1286.60	1025.83	79.73%

Abbreviations: QALY, quality-adjusted life year; DALY, disability-adjusted life year; HPV, human papilloma virus. ^a^ Alternative analysis including normative utilities.

#### Pneumococcal Vaccine Additional Scenarios

Similarly to the HPV model, different alternative scenarios were evaluated (EQ-5D weights replaced by 1-disability weight, in the alternative analysis in order to reduce between countries variability in QALYs gained estimations. New QALYs gained estimations produced a difference in the DALY/QALY percentage ratio in a range of -1% to +16% comparing against the base cases analysis. Table A6 shows new estimations using 1-disability weights as EQ-5D QALY weights. Since these changes were small, lower differences on threshold analysis using both QALYs gained and DALYs avoided for Chile and UK were still found when 3.5% discount rate and age weighting were used (see Figure A6). For Argentina, the lowest difference was found when 3.5% discount rate without age weighting analysis was performed.

**Table A6:** Incremental Utility Comparing Conjugated Pneumococcal Vaccination Against no Vaccination (Per 100 000 subjects)^a^

**Country**	**QALYs Gained**	**DALYs Avoided**	**DALY/QALY Percentage Ratio**
**Synflorix vs. no Vaccination**
**Without Discount, With Age Weighting**			
Argentina	16.03	20.27	126.44%
Chile	32.24	40.10	124.36%
UK	20.48	22.29	108.85%
**3.5% Discount Rate With Age Weighting**			
Argentina	6.39	7.25	113.46%
Chile	15.75	15.72	99.79%
UK	13.27	13.14	99.02%
**Without Discount, Without Age Weighting**			
Argentina	16.03	19.22	119.90%
Chile	32.24	40.00	124.05%
UK	20.48	28.95	141.36%
**3.5% Discount Rate Without Age Weighting**			
Argentina	6.39	6.87	107.55%
Chile	13.58	16.39	120.64%
UK	12.32	17.36	140.89%

Abbreviations: QALY, quality-adjusted life year; DALY, disability-adjusted life year. ^a^ Alternative analysis QALY coefficients = 1-disability weight.

Several alternative analyses were carried-out: excluding normative utilities from all models, using local life expectancies to DALYs estimations, using a same population structure and excluding herd protection from the analysis. As expected in the alternative analysis using normative utilities, QALYs gained increased, reducing the DALY/QALY percentage ratio in all countries (see Table A7). Using of local life expectancies in each country produced a DALYs avoided estimations reduction between -8% to -1% (see Table A8). Using the same population structure to the other countries produced a change on DALY/QALY percentage ratio ranged from -1% to +11% (see Table A9). Finally, the without herd immunity analysis produced the bigger differences between countries related to changes on the DALY/QALY percentage ratio. Meanwhile negligible changes were observed for Argentina, DALY/QALY percentage ratio was reduced for more than 50% in some cases for UK (see Table A9). As exclusion of herd immunity of models reduced the effect of vaccination only to less of 10 years old populations, age range used for economic evaluations could explain (at least in part) differences between DALYs and QALYs. Decision impact analyses for these alternative scenarios are shown in the Appendix, Figures A7 to A12.

**Table A7:** Incremental Utility Comparing Synflorix^®^ Vaccination Against no Vaccination (Per 100 000 Women)^a^

**Country**	**QALYs Gained**	**DALYs Avoided**	**DALY/QALY Percentage Ratio**
**Synflorix vs. no Vaccination**
**Without Discount, With Age Weighting**			
Argentina	18.13	20.27	111.83%
Chile	41.54	40.10	96.52%
UK	26.60	22.29	83.81%
**3.5% Discount Rate With Age Weighting**			
Argentina	7.36	7.25	98.55%
Chile	17.95	15.72	87.57%
UK	16.31	13.14	80.55%
**Without Discount, Without Age Weighting**			
Argentina	18.13	19.22	106.05%
Chile	41.54	40.00	96.29%
UK	26.60	28.95	108.85%
**3.5% Discount Rate Without Age Weighting**			
Argentina	7.36	6.87	93.42%
Chile	17.95	16.39	91.28%
UK	12.32	17.36	140.89%

Abbreviations: QALY, quality-adjusted life year; DALY, disability-adjusted life year. ^a^ Alternative analysis without normative utilities.

**Table A8:** Incremental Utility Comparing Synflorix^®^ Vaccination Against no Vaccination (Per 100 000 Women)^a^

**Country**	**QALYs Gained**	**DALYs Avoided**	**DALY/QALY Percentage Ratio**
**Synflorix vs. no Vaccination**
**Without Discount, With Age Weighting**			
Argentina	16.50	19.38	117.46%
Chile	35.84	39.31	109.68%
UK	21.47	21.93	102.15%
**3.5% Discount Rate With Age Weighting**			
Argentina	6.95	7.19	103.46%
Chile	15.75	15.57	98.84%
UK	13.20	13.05	98.88%
**Without Discount, Without Age Weighting**			
Argentina	16.50	17.66	107.05%
Chile	35.84	38.48	107.37%
UK	21.47	28.24	131.52%
**3.5% Discount Rate Without Age Weighting**			
Argentina	6.95	6.76	97.31%
Chile	15.75	16.07	101.99%
UK	13.20	17.20	130.34%

Abbreviations: QALY, quality-adjusted life year; DALY, disability-adjusted life year. ^a^ Alternative analysis using local life expectancies.

**Table A9:** Incremental Utility Comparing Synflorix^®^ Vaccination Against no Vaccination (Per 100 000 Women)^a^

**Country**	**QALYs Gained**	**DALYs Avoided**	**DALY/QALY Percentage Ratio**
**Synflorix vs. no Vaccination**
**Without Discount, With Age Weighting**			
Argentina	11.48	14.04	122.30%
Chile	31.18	34.66	111.17%
UK	21.47	22.29	103.83%
**3.5% Discount Rate With Age Weighting**			
Argentina	4.89	5.06	103.59%
Chile	14.57	14.60	100.23%
UK	13.20	13.14	99.55%
**Without Discount, Without Age Weighting**			
Argentina	11.48	13.38	116.61%
Chile	31.18	36.74	117.82%
UK	21.47	28.95	134.85%
**3.5% Discount Rate Without Age Weighting**			
Argentina	4.89	4.86	99.37%
Chile	14.57	16.82	115.49%
UK	13.20	17.36	131.58%

Abbreviations: QALY, quality-adjusted life year; DALY, disability-adjusted life year. ^a^ Alternative analysis using UK population structure.

A special analysis on two selected variables related to acute otitis media (AOM) was performed since variables related to this condition showed a higher impact on final cost-effectiveness ratio in the sensitivity analysis of the original models. In this sense, proportion of Streptococcus pneumoniae in AOM and AOM incidence were increased in a 20% in each country. Changes in QALYs gained and DALYs avoided estimations, as well as DALY/QALY percentage ratio and threshold analyses were negligible (lower of 1% for all countries), as compared against the base case analysis (see Tables A10 and A11).

**Table A10:** Incremental Utility Comparing Synflorix^®^ Vaccination Against no Vaccination (Per 100 000 Women)^a^

**Country**	**QALYs Gained**	**DALYs Avoided**	**DALY/QALY Percentage Ratio**
**Synflorix vs. no Vaccination**			
**Without Discount, With Age Weighting**			
Argentina	16.56	20.27	122.40%
Chile	35.89	40.10	111.71%
UK	21.54	22.29	103.49%
**3.5% Discount Rate With Age Weighting**			
Argentina	7.01	7.25	103.47%
Chile	15.81	15.72	99.43%
UK	13.27	13.14	99.02%
**Without Discount, Without Age Weighting**			
Argentina	16.56	19.23	116.11%
Chile	35.89	40.00	111.45%
UK	21.54	28.96	134.43%
**3.5% Discount Rate Without Age Weighting**			
Argentina	7.01	6.88	98.17%
Chile	15.81	16.39	103.67%
UK	13.27	17.37	130.91%

Abbreviations: QALY, quality-adjusted life year; DALY, disability-adjusted life year. ^a^ Alternative analysis AOM *Streptococcus pneumoniae* prevalence +20%.

**Table A11:** Incremental Utility Comparing Synflorix^®^ Vaccination Against no Vaccination (Per 100 000 Women)^a^

**Country**	**QALYs Gained**	**DALYs Avoided**	**DALY/QALY Percentage Ratio**
**Synflorix vs. no Vaccination**
**Without Discount, With Age Weighting**			
Argentina	16.59	20.27	122.17%
Chile	35.93	40.10	111.60%
UK	21.61	22.30	103.17%
**3.5% Discount Rate With Age Weighting**			
Argentina	7.04	7.26	103.01%
Chile	15.85	15.72	99.21%
UK	13.34	13.14	98.53%
**Without Discount, Without Age Weighting**			
Argentina	16.59	19.24	115.91%
Chile	35.93	40.01	111.34%
UK	21.61	28.96	134.04%
**3.5% Discount Rate Without Age Weighting**			
Argentina	7.04	6.89	97.78%
Chile	15.85	16.39	103.46%
UK	13.34	17.38	130.29%

Abbreviations: QALY, quality-adjusted life year; DALY, disability-adjusted life year. ^a^ Alternative analysis AOM incidence +20%.
